# Cyclic AMP Signaling: A Molecular Determinant of Peripheral Nerve Regeneration

**DOI:** 10.1155/2014/651625

**Published:** 2014-08-07

**Authors:** Eric P. Knott, Mazen Assi, Damien D. Pearse

**Affiliations:** ^1^The Miami Project to Cure Paralysis, The Miller School of Medicine at the University of Miami, Miami, FL 33136, USA; ^2^Herbert Wertheim College of Medicine, Florida International University, Miami, FL 33199, USA; ^3^The Department of Neurological Surgery, The Neuroscience Program, The Interdisciplinary Stem Cell Institute, The Miller School of Medicine at the University of Miami, Miami, FL 33136, USA

## Abstract

Disruption of axonal integrity during injury to the peripheral nerve system (PNS) sets into motion a cascade of responses that includes inflammation, Schwann cell mobilization, and the degeneration of the nerve fibers distal to the injury site. Yet, the injured PNS differentiates itself from the injured central nervous system (CNS) in its remarkable capacity for self-recovery, which, depending upon the length and type of nerve injury, involves a series of molecular events in both the injured neuron and associated Schwann cells that leads to axon regeneration, remyelination repair, and functional restitution. Herein we discuss the essential function of the second messenger, cyclic adenosine monophosphate (cyclic AMP), in the PNS repair process, highlighting the important role the conditioning lesion paradigm has played in understanding the mechanism(s) by which cyclic AMP exerts its proregenerative action. Furthermore, we review the studies that have therapeutically targeted cyclic AMP to enhance endogenous nerve repair.

## 1. Introduction

Injury to the peripheral nerve system (PNS), leading to motor, sensory, and/or autonomic functional loss, is not uncommon yet remains a challenge for surgeons. Peripheral nerves (PNs) can experience injury by way of stretch, laceration, compression and mechanical deformation, with the regenerative capacity of the nerves under each circumstance varying accordingly [1]. Unlike injury to the central nervous system (CNS), however, injury to the PNS induces a gene expression program that, in many cases, ultimately leads to self-recovery through axon regeneration and reconnection. With rare exception, PNs will maintain accuracy and reproducibility of connections after crush injury [2]. Yet, in the event of complete nerve laceration, the fidelity of axon regeneration is no longer guaranteed. In this case, the current “gold standard” of treatment requires that the space between nerve endings, also commonly referred to as the “nerve gap,” either be bridged or filled in with an entirely new distal pathway. Currently, surgical management of the gap includes the placement of a nerve autograft, nerve conduit, acellular nerve allograft, and for more severe and/or proximal nerve injuries, cellular nerve allografts and nerve transfers. Each surgical management strategy attempts in its own way to create a more ideal conduit by which the peripheral axons can return to their denervated targets and form functional synapses—a necessity for the restoration of function. Challenges to achieving full recovery in such a paradigm arise primarily due to the nature of the unilateral approach to therapy. In only targeting the extrinsic variables of regeneration, the success of the PN repair process becomes dependent primarily upon several potentially uncontrollable variables, such as the time-delay between injury and graft placement, the age of the patient, and the distance separating the nerve endings, rather than on the efficacy of the healing modalities available [1].

Over time, our understanding of the PN regeneration process has increased significantly. We now know that injured axons form proximal regenerative buds that, in a process largely governed by factors produced by Schwann cells (SCs), sprout and grow toward their distal targets. Furthermore, the importance of degeneration as the rate limiting step in the process of recovery has also been elucidated. After PNS injury there is exponential migration of microglia and macrophages to the lesion site for the purpose of removing debris [3]. This process clears the path for the growing axons. Once the debris is cleared, the proximal end of the injured axon sprouts regenerative buds. In the meantime, Wallerian degeneration occurs at the distal end of the injured axons, a process that includes degeneration of the axons and myelin, but not the endoneurium, which later serves as a conduit to direct axon growth back to their correct targets. The molecular mechanisms governing these responses have been a major focus of investigation over the last decade, producing a greater understanding of the signaling events in both neuron and glia that govern successful regeneration as well as offering novel targets for the development of therapeutic interventions (reviewed in [1, 4, 5]). A primary mediator of this intrinsic growth response of peripheral axons [6] is the second messenger, cyclic adenosine monophosphate (cyclic AMP), which is essential to PNS regeneration for both axon and SC responses.

## 2. Neuronally Expressed Cyclic AMP Signaling Intermediaries Involved in Nerve Regeneration

Injury to the PNS switches the neuron's function from the provision of neurotransmission to the musculature back to its developmental role of axon growth [7]. After the PN is injured, its cytoplasm is exposed to the extracellular environment, permitting calcium and sodium ions to freely flow into the axon through the ruptured plasma membrane. The unregulated flow of ions alters the membrane potential such that it becomes capable of generating a multitude of action potentials at the site of injury. These action potentials propagate in a retrograde manner to the cell body where the discharge promotes another influx of calcium through voltage-dependent ion channels. The influx of calcium at the site of axotomy and through voltage-gated calcium channels in turn promotes the activation of a variety of proteins. This activation continues temporally as subsequent waves of stimulating signals from the injured axons and from associated glial cells are retrogradely relayed to the neuronal somata. There has been work to show that this response to injury is quite different between the PNS and CNS and thus may account for the differences in the regenerative capabilities of the two systems [8].

**Figure 1:**
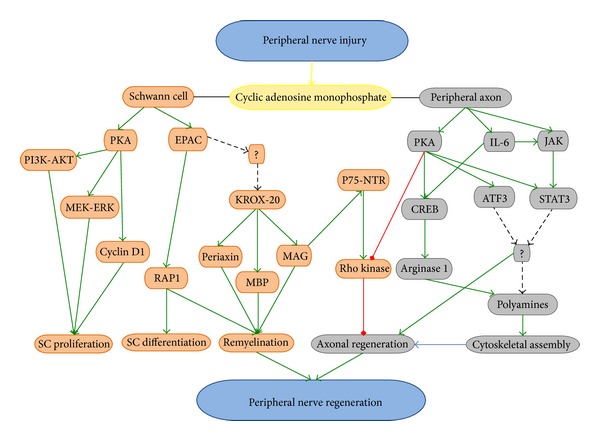
Following PN injury, cyclic AMP is involved in a variety of positive (green line), inhibitory (red line), and as yet to be identified (dashed line) signaling mechanisms within the injured neurons and their accompanying glia that culminates in PN regeneration.

One of the proteins activated by these signals is membrane-bound adenylyl cyclase, which converts adenosine triphosphate (ATP) to the second messenger, cyclic AMP [9, 10]. The production of cyclic AMP alters the physiology of the neuron, changing its function from one of transmission to one of growth through a series of downstream effectors (Figure 1). The effect of cyclic AMP to alter the intrinsic capacity of neurons to regenerate their axons can be recapitulated by the injection of a cyclic AMP synthetic analog, such as dibutyryl-cyclic AMP (db-cyclic AMP), into the dorsal root ganglion [11, 12]. It has been shown that the transcription-dependent effects of cyclic AMP on regeneration occur through both PKA-dependent [13] and PKA-independent signaling, via cyclic AMP activation of the cytokine interleukin 6 (IL-6) gene [14]. The PKA-independent pathway through IL-6 has been shown to involve IL-6 binding to the IL-6R and gp130 receptor and coreceptor, respectively. Receptor binding activates the Janus-Activated Kinase (JAK) family of tyrosine kinases [15]. JAK activation by IL-6 can allow neurites to overcome growth inhibition in response to myelin-associated glycoprotein (MAG) and myelin. The ultimate transcriptional target of IL-6 signaling is the transcription factor, signal transducer and activator of transcription 3 (STAT-3) [16], hypothesized to allow the transmission of retrograde signals from the axotomized axon to the nucleus to induce gene programs involved in neuronal survival and regeneration after nerve injury [17]. The necessity of IL-6 for cyclic AMP-mediated axonal regeneration, however, remains unclear, as blocking IL-6 signaling produces no effect on the ability of cyclic AMP to overcome axon growth inhibition by myelin inhibitors [14]. This work demonstrated that IL-6 was sufficient to overcome myelin inhibition of axon growth yet not necessary for the effects of cyclic AMP. However, Cafferty et al. reported that, following a conditioning lesion in IL-6 knock-out animals, no dorsal column axonal regeneration occurs, thus highlighting the importance of IL-6 as an effector of cyclic AMP in neuroregeneration [18].

The PKA-dependent pathway involved in cyclic AMP-mediated axon growth over inhibitory substrates, on the other hand, requires downstream activation of several key transcription factors, cyclic AMP responsive element binding protein (CREB), activating transcription factor type III (ATF-3), and STAT-3 [19–22]. Both STAT-3 and ATF-3 have been shown to influence DRG neurite regeneration and elongation. ATF-3 has demonstrated proregenerative qualities when neurons are cultured on permissive substrates, such as laminin [21, 23, 24]. ATF-3, however, is not sufficient to overcome the inhibitory effects of myelin nor is it able to promote central axonal regeneration in the spinal cord* in vivo* [21]. In contrast, the activation of CREB alone has been shown to be sufficient for overcoming myelin's inhibition of neurite outgrowth [13]. While activation of the intrinsic growth capacity of neurons through cyclic AMP occurs at the transcriptional level to promote axonal elongation, the regenerative effects of cyclic AMP and PKA can also affect axonal growth through direct effects on cytoskeletal behavior [13, 25].

PKA can directly alter cytoskeletal effectors at the axon to stimulate growth by way of disinhibition. Thus far, three major inhibitors of neurite growth have been identified in myelin, Nogo, Ogmp, and MAG, the last of which appears to be the main inhibitory component of PN myelin [26–28]. It has been demonstrated that MAG activates the small GTPase, Rho-A, in a p75^NTR^ dependent manner [29]. The Rho-A GTPase signals through activation of Rho-associated kinase (ROCK) to inhibit axon cytoskeletal assembly [30, 31]. While studies to date in nonneuronal cells have suggested that PKA may phosphorylate Rho-A and negatively regulate its activity, it is unclear whether this mechanism occurs in cyclic AMP-PKA-mediated relief of neurite outgrowth inhibition on myelin. It has been shown, however, that after nerve injury, the activation of GTP-bound Rho-A, which is normally undetectable in intact ganglia, is dramatically upregulated in peripheral neurons [30]. Furthermore, ROCK inhibition using fasudil, a selective RhoA/Rho kinase (ROCK) inhibitor, after nerve injury, has been shown to facilitate repair as detected by way of amplitude measurements of distally evoked compound muscle action potentials, which were faster after axonal injury in mice treated with fasudil [30, 31]. The transcriptional-dependent mechanisms of cyclic AMP-PKA-induced axon growth have been also shown to regulate cytoskeletal assembly through CREB activation of the gene* arginase-1* and ensuing polyamine synthesis [25]. Wolff [32] demonstrated that the rate and extent of microtubule assembly from nervous system tubulin are enhanced by oligocations including polyamines. Polyamines, through* arginase-1,* promote neuronal cytoskeletal assembly via tubulin stimulation [32]. Arginase 1 is upregulated after a peripheral lesion and is a rate-limiting enzyme in the synthesis of the polyamines putrescine, spermidine, and spermine, which together are essential for axon cytoskeleton assembly [25]. The importance of polyamine synthesis in cyclic AMP-mediated neurite growth on MAG and myelin has been demonstrated in inhibition studies, while overexpression of arginase has been shown to overcome neurite growth inhibition by MAG and myelin [25].

## 3. The Molecular Role of Cyclic AMP in Schwann Cell Responses during PN Repair

After PN injury, Schwann cells (SCs) undergo a series of cellular changes that include dedifferentiation, proliferation, and then differentiation back to a myelinating phenotype. The transition between these stages and the functionality of the SCs within each rely heavily on cyclic AMP signaling. Initial physical damage to the PN triggers a cellular response of immune cells and SCs in the distal nerve termed Wallerian degeneration. During this process the SCs lose their myelin sheaths and undergo dedifferentiation. This allows the SCs to subsequently proliferate in response to signals from the regenerating axons to ensure that sufficient numbers of SCs are generated to replace those lost during injury and to allow them to mediate remyelination repair. The initial changes in SCs as they undergo dedifferentiation, their proliferation and association with axons, and their differentiation and remyelination of axons have all been shown to involve cyclic AMP signaling [33–35].

Studies have demonstrated that cyclic AMP is important for SC replication through its positive interaction with receptor tyrosine kinases (RTKs) [35, 36]. Platelet-derived growth factor (PDGF), basic fibroblast growth factor (bFGF), insulin-like growth factor (IGF), and regenerating protein 1 (Reg-1) all promote SC proliferation, but only when they are added in conjunction with cyclic AMP or an agent that promotes elevated cyclic AMP levels, such as forskolin [35, 37, 38]. Members of the neuregulin family of growth factors, on the other hand, function in a self-reliant manner to promote the proliferation of SCs [33, 39], but do so only because they promote the accumulation of endogenous cyclic AMP within the cell.

The cyclic AMP-dependent processes involved in the dedifferentiation and proliferation of SCs occur either through its direct activation of PKA, or due to its role as a “gating” molecule, by regulating the flow of signals through other pathways. Kim and colleagues (2001) showed that when SCs are cultured in PDGF-free medium, they become growth-arrested, with a diploid (G1) content of DNA. Furthermore, although PDGF is a positive signal for DNA replication and division of SCs, PDGF-treated SCs do not progress through the G1 phase of the cell cycle toward S phase until they are exposed to cyclic AMP. In the same study it was demonstrated that PKA activity in SCs undergoing proliferation maintains the expression of cyclin D1 after the initial mitogenic cue has been given by a receptor tyrosine kinase. Importantly, the proliferation of mature SCs is strictly dependent upon the expression of cyclin D1 [40]; however, the mechanism by which forskolin sustains and reinitiates cyclin D1 was not elucidated in this work. In contrast, neuregulin-induced SC proliferation, in which this signal alone increases cyclic AMP, has been reported by Monje and colleagues (2008) to involve a more complex interaction in which the effect of cyclic AMP on S-phase entry relies also on the ability of the second messenger to enhance the effects of neuregulin-stimulated MEK-ERK and PI3K-Akt activation, both of which are required for progression of the cell cycle. The activation of PKA is still ultimately required for triggering S-phase entry of SCs by concomitantly enhancing the ligand-dependent tyrosine phosphorylation and activation of the neuregulin coreceptor, ErbB2-ErbB3 [34].

How cyclic AMP regulates the SC's response to injury is unique, however, in that cyclic AMP can act as both a mitogen and as a differentiation signal. In addition to promoting SC proliferation, cyclic AMP signaling also mediates the exit of SCs from the cell cycle by way of their transformation into a premyelinating phenotype [36]. Elevation of cyclic AMP promotes the expression of the transcription factor Krox-20 (otherwise known as Egr2), which drives the expression of an array of myelin-related proteins and lipids, as well as transcription factors including Oct-6 and NF-*κ*B [41–44]. Although Krox-20 null SCs have been shown to express the early myelin marker, myelin-associated glycoprotein (MAG), they fail to ensheathe axons and do not upregulate myelin basic protein (MBP) [41]. Mirsky and coworkers [45] showed that, in the presence of cyclic AMP, Krox-20 null SCs still express the protein periaxin, which is required for the maintenance of myelin [45]. Furthermore, periaxin is expressed in these mice after PN injury, indicating that important myelin-associated genes can be regulated by cyclic AMP through both Krox-20-dependent and -independent mechanisms.

This seeming contradictory function of cyclic AMP as both a mitogen and in promoting differentiation has been suggested to result from the two main downstream effector pathways of cyclic AMP, PKA and the exchange protein activated by cyclic AMP (EPAC) [46]. This question was recently examined in studies by Bacallao and Monje (2013) in which they showed that SC dedifferentiation and proliferation required PKA activation, not EPAC, while differentiation into myelin-forming cells involved EPAC rather than PKA. EPAC is able to directly transduce cyclic AMP signals through its ability to act as a guanine nucleotide exchange factor for the small GTP-binding protein Rap1. In this work, however, EPAC activation alone was demonstrated to be sufficient neither for a full differentiating response, nor for the expression of Krox-20, suggesting that more work needs to be done to identify the molecular effectors of cyclic AMP in the initiation of SC differentiation [47].

## 4. The Conditioning Lesion as a Paradigm for Regenerative Strategies

Following PN injury, functionally meaningful regeneration requires that the injured axons regrow to reinnervate their denervated targets through the formation of functional synapses and that these axons are then remyelinated to allow for proper axon conduction. However, current therapeutic strategies, in particular surgical interventions, are often insufficient. One avenue for identifying intrinsic-based strategies that may enhance the regenerative potential of peripheral neurons as an adjuvant therapy after complex injuries has been to study the underlying mechanisms involved in the conditioning PN lesion, or simply the conditioning lesion. This powerful tool has provided a greater understanding of the molecular mechanisms responsible for the differences in regenerative potential between the two axonal branches of the bipolar dorsal root ganglion neuron. The conditioning lesion describes the phenomenon of the enhanced regenerative potential that is acquired by the central branch of a dorsal root ganglion neuron after the peripheral branch is injured, through the activation of specific intrinsic growth signaling programs. The central branch can then regenerate into and beyond a lesion when made in the CNS, even overcoming the inhibitory environment of the spinal cord [48–50]. In 2002, Cai et al. demonstrated that one of the key components of the conditioning lesion was to raise cyclic AMP levels in the injured neuronal somata, allowing the axons to then be able to overcome MAG inhibition of elongation by triggering a transcription-dependent rise in regeneration-associated genes [25], such as the expression of arginase 1 [51].

Soon after cyclic AMP signaling was implicated as a main effector in the conditioning paradigm, it was demonstrated that intraganglionic injection of a membrane permeable cyclic AMP analog, dibutyryl cyclic AMP (db-cyclic AMP), could mimic the effects of a conditioning lesion and that this effect occurred through activation of PKA [20, 52]. Similar to this phenomenon in PN injury, intraocular injection of db-cyclic AMP has been shown to enable optic nerve growth after optic nerve injury, implicating cyclic AMP signaling as an important event in the induction of intrinsic axon growth programs in both peripheral and central neuron populations [48, 53–55]. Although methods to raise cyclic AMP by the injection of an analog can replicate to some extent the conditioning paradigm, it does not completely provide the same degree of regenerative benefit and does not produce sustained axon regeneration when given as a single injection [52, 53, 56]. This lack of sustained regenerative capacity illustrates the need for a better understanding of the downstream signaling, pathway crosstalk, and the timing and subcellular compartmentalization of cyclic AMP in stimulating intrinsic axon growth initiation and elongation.

Being a second messenger important in many biological processes, it is not surprising that cyclic AMP is involved in a variety of signaling pathways that mediate PN regeneration. However, it is in the role that cyclic AMP plays as a simultaneous mediator of both the intrinsic ability of axons to regenerate as well as releasing environmental growth inhibition, in both the PNS and CNS, that the activation of the cyclic AMP pathway in the PNS is unique. With roles for cyclic AMP also at the axonal growth cone and within SCs at the periphery, which are important for guiding and remyelinating the regrowing axon, integrating these responses and maximizing them for the greatest degree of PN repair remain important therapeutic considerations.

## 5. Targeting the Cyclic AMP Pathway for PN Regeneration

Currently, the main focus for therapeutics in PN repair is the use of artificial nerve guidance channels to match or exceed the performance of the gold standard, an autograft. To date, the use of molecular or pharmacological methods to increase the intrinsic capacity of injured PNs to regenerate has remained within the arena of experimental research. In animal PN lesion models, enhancing the production or inhibiting the metabolism of cyclic AMP have been employed for therapeutic effect. Adenylate cyclase activators, cyclic AMP analogs, and phosphodiesterase (PDE) inhibitors have all been employed to promote PN repair using primarily pharmacological agents. In 1987, Kilmer and Carlsen first demonstrated that the daily injection or delivery of forskolin through an implanted osmotic pump produced a sustained 40% increase in the rate of sensory nerve regeneration and an approximately 40-fold greater elevation in neuronal cyclic AMP than an equimolar concentration of a control, isoprenaline, after freeze-lesioning of the sciatic nerve. Three years later the same group tested the hypothesis that cyclic AMP modulates nerve regeneration in mammals by comparing the effects of chronically infused forskolin with that of infused db-cyclic AMP, 8-bromo cyclic AMP, or the PDE inhibitor, theophylline, in hamsters [57]. The results from these studies demonstrated that all methods targeting cyclic AMP elevation were able to enhance regeneration, though forskolin and 8-bromo cyclic AMP had the most profound effect on axonal elongation, and theophylline produced the largest decrease in the initiation time required for neurite sprouting. Interestingly the effect of theophylline was mirrored by caffeine, a methylxanthine that can increase intracellular calcium but also has a limited ability to inhibit PDEs [57]. However, in these studies the relative levels of increased cyclic AMP among the different approaches were not investigated and thus it is not clear whether such an effect may have accounted for their different levels of potency on PN regeneration.

In later studies it was found that while forskolin alone was sufficient to increase cyclic AMP levels in normal nerve and following nerve crush during the period of axon regeneration, it could not do so if the nerve had been transected [58]. Following nerve transection, cyclic AMP could only be elevated by the combination of forskolin (to activate adenylyl cyclase) and 3-isobutyl-1-methylxanthine (IBMX), a general inhibitor of PDEs, which hydrolyze cyclic AMP. It was suggested in this work that PDE inhibition was necessary in order to elevate cyclic AMP levels in nonmyelinating nerves, which exhibited a robust induction of PDE activity [58]. Subsequent experiments demonstrated that the PDE4 inhibitor Rolipram was very effective in enhancing PN regeneration after injury and that not only was Rolipram treatment alone sufficient for promoting PN regeneration, but it also accelerated the reinnervation of denervated skeletal muscles [59, 60]. The effect of cyclic AMP on PN regeneration appears to involve intermediaries both directly downstream of its effector pathways, PKA and EPAC, as well as from crosstalk between cyclic AMP and other signaling cascades, such as the Rho-A/ROCK pathway. Studies have shown that one growth promoting effect of cyclic AMP is to antagonize Rho-A GTPase signaling, which has been implicated in growth cone collapse as well as in the intracellular transmission of signals from axon growth inhibitory molecules, such as myelin [52]. While the involvement of the Rho-A GTPase and downstream ROCK in axon growth inhibition has been best characterized in models of CNS injury, such as spinal cord injury [52], work in PN regeneration failure has also focused on this pathway. Cheng and colleagues [30] reported that the activation of GTP-bound Rho-A, which is normally undetectable in intact ganglia, was dramatically upregulated in both neuronal soma and axons after injury. Later, Huelsenbeck et al. [61] employed* Clostridium botulinum* C3-exoenzyme to nonenzymatically downregulate active Rho-A after PN crush. Inhibition of Rho-A would promote axon growth via disinhibition of cytoskeletal assembly mediated by ROCK. They demonstrated that daily or a one-time topical application of a 26-amino-acid fragment of C3 after PN crush or in a nerve autotransplantation paradigm, respectively, in rat resulted in improved axonal elongation and faster motor recovery [61]. Interestingly, a study by Auer and colleagues [62], using a C3 mutant exoenzyme that lacked RhoA inhibitory activity, showed that this deficient C3 could also promote axonal growth, suggesting that effectors other than Rho-A may be involved in such responses. Other studies employing the ROCK inhibitor Fasudil have shown that this compound can promote PN repair after injury as measured by increased amplitude recordings of distally evoked compound muscle action potentials [30, 31].

Although much of the work aimed at increasing cyclic AMP after PN injury has focused on pharmacological approaches, alternative strategies to enhancing cyclic AMP for PN repair do exist, in particular the use of electrical stimulation. In the 1980s it was reported that low frequency electrical stimulation of PNs after crush injury could accelerate the return of reflex foot withdrawal and contractile force in reinnervated leg muscles [63, 64]. Al-Majed and colleagues later demonstrated that electrical stimulation of the lacerated and graft repaired rat femoral nerve using the same low frequency stimulation could accelerate sensory axon growth and direct axons specifically into their correct sensory nerve pathways [65]. In later work they would show that such accelerated growth in response to low frequency electrical stimulation required cyclic AMP and PKA activation [66].

## 6. Conclusions and the Future of Targeting Cyclic AMP for PN Repair

In summary, following PN injury, cyclic AMP serves a crucial role in activating many of the signaling pathways that ultimately produce functional nerve regeneration. This second messenger, by way of various transcription-dependent pathways, promotes axonal growth and myelination, as well as SC proliferation and differentiation. Transcription-dependent pathways leading to axonal growth depend largely on activation of CREB and the inhibition of cytoskeletal inhibitors by PKA. While SC proliferation has been found to be PKA dependent and differentiation and myelination have been found to be EPAC dependent, both these pathways are nevertheless ultimately cyclic AMP-activation dependent, in what are no longer considered opposing pathways.

Pharmacological and nonpharmacological strategies targeting cyclic AMP and its upstream or downstream effectors have shown promise for management of PN injury. Such utilization of cyclic AMP-dependent pathways to enhance PNS recovery would complement the extrinsic approach of surgical modalities utilized in clinical practice today, providing a more holistic and potentially efficacious therapeutic approach to neuroregeneration of PN injury.
